# Impact of the COVID-19 Pandemic on the Incidence of Notifiable Infectious Diseases in China Based on SARIMA Models Between 2013 and 2021

**DOI:** 10.1007/s44197-024-00273-x

**Published:** 2024-07-30

**Authors:** Jingwen Liu, Wu Zeng, Chao Zhuo, Yu Liu, Lei Zhu, Guanyang Zou

**Affiliations:** 1 School of Public Health and Management, Guangzhou University of Chinese Medicine, Guangzhou, China; 2 Department of Global Health, School of Health, Georgetown University, Washington, DC USA; 3 State Key Laboratory of Respiratory Disease, First Affiliated Hospital of Guangzhou Medical University, Guangzhou, China; 4 Office of Academic Affairs, Guangzhou University of Chinese Medicine, Guangzhou, China

**Keywords:** COVID-19, Public health, Epidemiology, Mathematical modelling, ARIMA

## Abstract

**Background:**

During the COVID-19 pandemic, the Chinese government implemented nationwide public health interventions to control its spread. However, the impact of these measures on other infectious diseases remains unclear.

**Methods:**

The incidence of three types of notifiable infectious diseases in China were analyzed between 2013 and 2021. The seasonal Mann-Kendall test and Mann-Kendall mutation test were employed to examine trends and mutations in the time series. Based on the counterfactual inference, historical incidence rates were employed to construct SARIMA models and predict incidence between January 2020 and December 2021. Differences between reported and predicted incidences during the pandemic were compared using the Mann-Whitney U test.

**Results:**

Between 2013 and 2019, the incidence rate of three types of notifiable infectious diseases fluctuated between 494.05/100,000 and 550.62/100,000. No discernible trend was observed for types A and B infectious diseases (Z = −1.344, *P* = 0.18). A significant upward trend was observed for type C infectious diseases (Z = 2.56, *P* = 0.01). In 2020, the overall incidence rate of three types of notifiable infectious diseases decreased to 367.08/100,000. Compared to predicted values, the reported incidence of three types of infectious diseases was, on average, 30.05% lower in 2020 and 16.58% lower in 2021.

**Conclusion:**

The public health interventions implemented during the pandemic had a positive consequence on the prevention and control of other infectious diseases, with a particularly notable effect on type C infectious diseases. Among the diseases with different transmission routes, respiratory diseases and gastrointestinal or enteroviral diseases decreased significantly.

**Supplementary Information:**

The online version contains supplementary material available at 10.1007/s44197-024-00273-x.

## Introduction

Detecting an outbreak of infectious disease is a cornerstone of public health to mitigate risk and prevent further transmission [1]. In December 2019, an outbreak of COVID-19 occurred in Wuhan, Hubei, China, with subsequent infections of different magnitude in different provinces and regions. On 11 March 2020, COVID-19 had spread globally, and the World Health Organization officially announced it as the second largest pandemic of the 21st century [2]. At the onset of the outbreak, the Chinese government classified COVID-19 as a type B infectious disease, yet managed it with protocols typically reserved for type A infectious diseases [3].

In response to the emergence of the COVID-19 pandemic, the Chinese government initiated a comprehensive nationwide vaccination program and implemented a series of robust public health interventions, including the use of masks, compulsory home quarantine, restriction of movement, and so on. These measures began with the lockdown of Wuhan on 23 January, when all provinces in the country activated the highest response level for public health emergencies [4]. Subsequently, varying degrees of prevention and control measures had also been introduced in countries around the world. The effects of such measures have been widely discussed. For instance, Barnett-Howell’s study revealed that prevention and control measures in low-income countries were less epidemiologically and socially valuable [5]. A study conducted in the United Kingdom found that the measures resulted in increased mortality from breast, colorectal, lung and oesophageal cancers, primarily due to changes in health-seeking behavior and the availability and accessibility of basic diagnostic services [6].

Despite their many disadvantages, prevention and control measures have been proven to be essential in controlling the spread of COVID-19 and other infectious diseases [3, 7, 8]. Luzhao Feng and colleagues, by constructing a time-series model, demonstrated that the implementation of such measures during the pandemic could effectively mitigate both seasonal and potential pandemic influenza [9]. A study by Olsen et al. also revealed a reduction in influenza activity in the United States, Australia, Chile, and South Africa during the COVID-19 pandemic [10]. Similarly, Zheng Zhao et al. developed a set of SARIMA models for hand, foot and mouth disease (HFMD) in 31 provincial capitals and municipalities in China and found that the disease control measures during the pandemic were associated with a 52.9% reduction in HFMD cases [11]. Furthermore, the incidence of a number of infectious diseases with different modes of transmission has also declined following the implementation of public health interventions [4, 12].

Understanding the impact of public health interventions implemented during the COVID-19 pandemic on different types of infectious diseases is essential for guiding the prevention and control of infectious diseases in the post-pandemic era [13, 14]. Previous studies, with a short period of data after Jan 2020, reported that public health interventions during the COVID-19 pandemic might have contributed to a reduction in the transmission of different types of notifiable infectious diseases in China [4, 15, 16]. Given the limited time series (until December 2020), the previous studies focused on a more immediate spill-over impact of COVID-19 control interventions on other infectious diseases after the outbreak. Comparative studies, with a longer period of post COVID-19 outbreak, are needed to enhance the understanding of not only the short-term but also medium-term impact of the COVID-19 outbreaks and interventions on different types of notifiable infectious diseases.

In this study, we collected data on the incidence of various infectious diseases in China between 2013 and 2021 with additional four quarters of data, in comparison with data from the previous studies. The data were published by the National Centre for Disease Control and Prevention. To quantify the impact of public health interventions on three types of notifiable infectious diseases, we developed time-series models to fit historical incidence rates and compared the reported incidence rates of each type of infectious disease in 2020–2021 with the predicted incidence rates under a counterfactual scenario of no COVID-19 pandemic and related public health interventions. The results of this study would improve our understanding of the effectiveness of public health interventions implemented during the COVID-19 outbreaks in mitigating different types of infectious diseases in China, and provide empirical evidence of behavior change during the pandemic on other infectious diseases.

## Materials and Methods

### Material Sources

Data on notifiable infectious diseases in China from January 2013 to December 2021 were collected from the official website of the Health Commission of the People’s Republic of China, excluding Hong Kong, Macao and Taiwan (http://www.nhc.gov.cn/jkj/s2907/new_list.shtml) [17]. These data are publicly available for research purposes and do not contain any personally identifiable information. Incidences were calculated using statistics from the previous year’s national resident population data, which were published by the National Bureau of Statistics. As the incidence rate for type A infectious diseases was extremely low, we combined the incidences for type A and type B infectious diseases in the analysis. Additionally, the study excluded influenza because of a widespread outbreak of influenza (type C infectious disease) in mainland China in December 2019. This was done to enhance the model’s accuracy and predictive power and to mitigate estimation bias.

### Classification of Infectious Diseases

The Law of the People’s Republic of China on Infectious Diseases categorises infectious diseases in China into three types (A, B, and C), based on their transmissibility and virulence ranking from high to low. Different types of infectious diseases will be subject to different levels of prevention and control measures. For example, type A infectious diseases, subject to the highest level of disease control measures, require compulsory isolation and treatment for patients, suspected patients and close contacts, and report the infectious disease cases within two hours of diagnosis, while most type B and all type C infectious diseases require to report cases within 24 hours [18]. Type A infectious diseases currently refer to plague and cholera. Type B infectious diseases consist of 27 infectious diseases, such as tuberculosis (TB), viral hepatitis, acquired immunodeficiency syndrome (AIDS), and rabies; and Type C infectious diseases consist of 11 infectious diseases, including HFMD, influenza, and mumps.

### Model Construction and Evaluation

#### ARIMA Model and SARIMA Model

To predict the incidence of different types infectious diseases, we used historical data from January 2013 to December 2019 tomake the prediction from 2020 to 2021 with an autoregressive integrated moving average (ARIMA) model. The predicted incidence was regarded as the scenario without COVID-19 impact. The ARIMA model is commonly used to predict the incidence of various types of infectious diseases [19–21], and the seasonal autoregressive integrated moving average (SARIMA) model has a better fitting and predictive effect for the incidence of infectious diseases with seasonal transmission patterns [22].

The models can be classified into various categories including ARIMA (p, d, q) and ARIMA (p, d, q) (P, D, Q) _(s)_, with ARIMA (p, d, q) (P, D, Q) _(s)_ representing the SARIMA representation of the model. In the ARIMA model, the letters p, d, and q represent the order of the time series autoregressive, differencing, and moving average components, respectively. Similarly, in the SARIMA model, the letters P, D, and Q represent the seasonal autoregressive, seasonal differencing, and seasonal moving average order components, respectively, and ‘s’ denotes the length of the periodicity. The models were constructed and evaluated using R version 4.3.2, and the auto.arima function in the‘forecast’ package automatically modelled and selected the optimal combination.

#### Stationarity Test and Model Selection

The monthly data from January 2013 to December 2019 (before the outbreak of COVID-19) were used as a training set to construct ARIMA models. The stationarity of the time series was tested by the augmented Dickey-Fuller (ADF) test. Non-stationary sequences were transformed into stationary sequences by differencing and exponential transformation. The best-fitted model was selected based on the minimum value of the Akaike information criterion (AIC). The model fit was measured by computing the Bayesian information criterion (BIC), Root mean square error (RMSE), Mean absolute error (MAE), Mean absolute percentage error (MAPE) and R^2^. A higher R^2^ and lower remaining statistics indicate a better fit of the model.

#### Model Validation and Prediction

To further validate the accuracy and reliability of the models, we used data from 2013 to 2018 as a training set to predict the incidence rates in 2019. The results were then evaluated by comparing the reported and predicted values and the MAPE values. The optimal model was used to predict the incidence rates from January 2020 to December 2021 as the counterfactual scenario without the influence of the COVID-19 pandemic, and then the difference between the reported values and the predicted values was used to assess the influence of the COVID-19 pandemic on incidence rates of three types of notifiable infectious diseases.

### Subgroup Analysis

Additional analyses were conducted for specific infectious diseases based on their transmission modes to provide a more detailed understanding of the impact of COVID-19 control measures. Specifically, we selected three diseases of high incidence or substantial concern from each of the following transmission categories: respiratory diseases, gastrointestinal or enteric viral diseases, sexually transmitted or blood-borne diseases, and vector-borne or zoonotic diseases.

### Data Collection and Statistical Analysis

Excel 2016 was utilised for storing the data and performing descriptive analyses (e.g. estimation of incidence) of three types of notifiable infectious diseases. The seasonal Mann-Kendall test was used to examine trends in the incidence of each type of infectious disease, and the Mann-Kendall mutation test was employed to detect sequence mutations [23]. SARIMA models were constructed and evaluated by using the auto.arima function in the'forecast' package of R version 4.3.2. SPSS 22.0 was used for the two-tailed non-parametric Mann-Whitney U test, with *P* < 0.05 indicating statistically significant differences.

## Result

### Profiles of Three Types of Notifiable Infectious Diseases in China, 2013–2021

Between 2013 and 2019, the average annual incidence of three types of notifiable infectious diseases was 525.27/100,000, with an average of 7,223,169 cases per year. The number of cases was the highest in 2018 with 7,653,990 cases reported. The incidence rates of three types of notifiable infectious diseases remained stable between 2013 and 2019. Similarity, the overall trend of types A and B infectious diseases exhibited a slight decline, from 270.96/100,000 in 2013 to 263.66/100,000 in 2019, with no significant fluctuations. The incidence rate of type C infectious diseases increased from 241.45/100,000 in 2013 to 263.31/100,000 in 2019. The results of the seasonal Mann-Kendall test suggested a significant upward trend for type C infectious diseases (Z = 2.56, *P* = 0.01), but no significant trend for types A and B infectious diseases (Z = −1.344, *P* = 0.18).

However, the results of the Mann-Kendall mutation test indicated a significant change in the incidence of both types A and B, and type C infectious diseases after 2019 (please refer to Appendix 1 for change point). Following the COVID-19 outbreak, the incidence of three types of infectious diseases decreased significantly to 367.08/100,000, with the incidence of type C infectious diseases decreasing from 263.31/100,000 in 2019 to 143.29/100,000 in 2020 and types A and B infectious diseases decreasing from 263.66/100,000 to 223.08/100,000 in the same period. The incidence of three types of infectious diseases in 2021 remained at a low level of 434.99/100,000, slightly higher than that in 2020, but lower than that in 2019. The detailed information can be found in Table 1.

**Table 1:** Number of infections and annual incidence rates of three types of notifiable infectious diseases(except COVID-19 and influenza), 2013–2021

Year	Types A and B		Type C		Types A, B and C	
	Cases	Incidence^a^ /100,000	Cases	Incidence /100,000	Cases	Incidence /100,000
2013	3,668,931	270.96	3,269,296	241.45	6,938,227	512.41
2014	3,587,215	263.63	3,942,040	289.70	7,529,255	553.33
2015	3,563,878	260.55	3,193,901	233.50	6,757,679	494.05
2016	3,470,910	252.71	3,714,901	270.47	7,185,811	523.18
2017	3,610,386	261.11	3,533,575	255.55	7,143,961	516.66
2018	3,664,034	263.58	3,989,956	287.03	7,653,990	550.62
2019	3,679,089	263.66	3,674,170	263.31	7,353,259	526.97
2020	3,133,258	223.80	2,006,114	143.29	5,139,372	367.08
2021	3,274,810	231.96	2,866,306	203.03	6,141,116	434.99

^a^ Population data were based on the national resident population at the end of the previous year, as published by the National Bureau of Statistics. The total population used to calculate infectious disease incidence rates from 2013 to 2021 was 1.354 billion, 1.361 billion, 1.368 billion, 1.373 billion, 1.383 billion, 1.390 billion, 1.395 billion, 1.400 billion, 1.412 billion respectively.

### Prediction and Comparison

#### Model Construction and Evaluation

In the process of model construction, due to the widespread outbreak of influenza epidemic in mainland China in 2019, the model fitting was effectively improved after the removal of influenza in this study (please refer to Appendix 2 for model constructions that include influenza). The results of the ADF test indicate that the sequence of types A, B, and C infectious diseases, types A and B, as well as type Cinfectious diseases were stationary (*P* = 0.01). The Ljung-Box test results showed that all of the aforementioned sequences were non-white noise series (*P* < 0.001). These results suggest that the sequences can be used in subsequent model construction. The models selected for types A, B and C infectious diseases, types A and B infectious disease and type C infectious diseases were ARIMA (1,0,0) (1,1,0) _(12)_ (model 1), ARIMA (2,1,1) (2,1,0) _(12)_ (model 2) and ARIMA (2,0,0) (1,1,0) _(12)_ (model 3), respectively. The R^2^ for models 1, 2, and 3 was 0.87, 0.82, and 0.90, respectively, indicating a good fit. The detailed information can be found in Table 2.

The results of the residual tests of the above three models showed that the residuals for all three models conformed to the white noise series (Model 1: χ² = 0.29, *P* = 0.59; Model 2: χ² = 0.02, *P* = 0.89; Model 3: χ² = 0.002, *P* = 0.96), suggesting that the residuals from the model followed a normal distribution and fluctuated around zero with no autocorrelation (please refer to Appendix 3 for normal Q-Q plot, residual ACF plot and residual PACF plot). The result of the model validation suggests that the predicted and reported values are relatively consistent, with the MAPE for the three models being 7.49%, 3.67%, and 13.77% respectively, indicating that all three models had good predictive performance (please refer to Appendix 4 for further details).

**Table 2:** Optimal ARIMA models for three types of notifiable infectious diseases, and evaluation of the model fit and diagnosis

Model ID	Model Type	ADF *P* value	AIC	BIC	Ljung-Box test		RMSE	MAE	MAPE	*R* ^2^
					Chi-square	*P* value				
Model_1(Types A B and C)	ARIMA (1,0,0) (1,1,0) _(12)_	0.01	410.42	417.25	43.19	< 0.001	3.53	2.59	6.12	0.87
Model_2 (Types A and B)	ARIMA (2,1,1) (2,1,0) _(12)_	0.01	209.86	223.44	19.28	< 0.001	0.84	0.60	2.83	0.82
Model_3 (Type C)	ARIMA (2,0,0) (1,1,0) _(12)_	0.01	382.97	392.07	44.22	< 0.001	2.85	2.12	10.42	0.90

### Empirical Validation

#### Overall Changes in Three Types of Notifiable Infectious Diseases Compared with Predictions

**Fig. 1:**
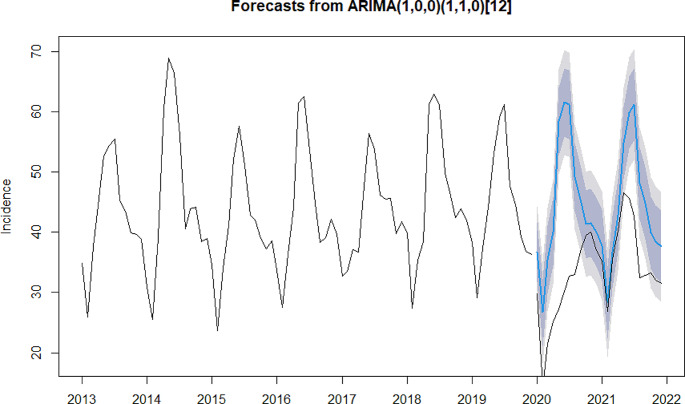
Difference between reported and predicted incidences for types A B and C notifiable infectious diseases. The black lines represent reported values. The blue line represents predicted values

The reported and predicted values for three types of notifiable infectious diseases and their 95% CI are shown in Fig. 1. In all 24 months of 2020 and 2021, the reported incidence was lower than the predicted value. In 2020, the difference between reported and predicted values was on average − 30.05% for three types of notifiable infectious disease (*P* < 0.05). The largest difference was observed in May 2020, with a difference of −53.34%, while the smallest difference was observed in November 2020, with a difference of −3.74%. In 2021, the difference was on average − 16.58% for three types of notifiable infectious disease (*P* < 0.05). From January to April 2021, the differences between the reported and predicted values were relatively minimal. However, from May 2021 onwards, the differences remained at a higher level. The detailed reported and predicted values are shown in Appendix 5.

#### Changes in Different Types of Notifiable Infectious Diseases Compared with Predictions

**Fig. 2:**
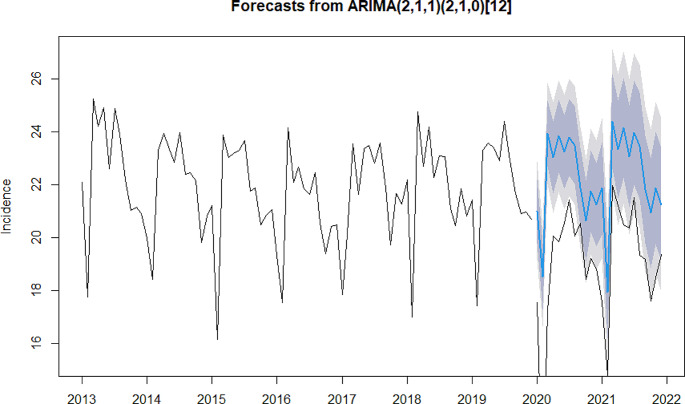
Difference between reported and predicted incidences for types A and B notifiable infectious disease. The black lines represent reported values. The blue line represents predicted values

**Fig. 3:**
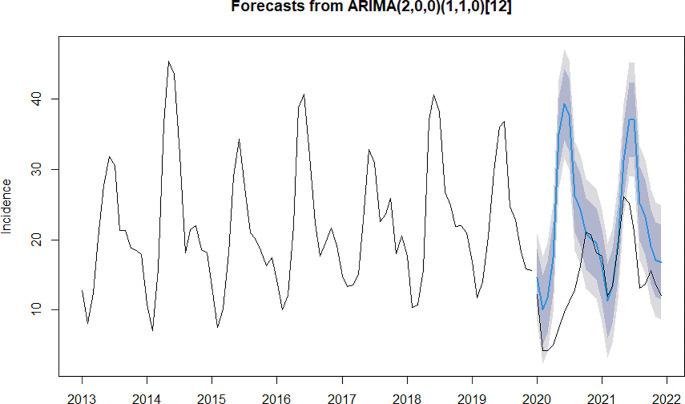
Difference between reported and predicted incidences for type C notifiable infectious disease. The black lines represent reported values. The blue line represents predicted values

As shown in Figs. 2 and 3, in 2020, the reported values for types A and B infectious diseases and type C infectious diseases were all lower than the model predictions. The difference was on average − 16.35% for types A and B infectious diseases (*P* < 0.05), and − 43.40% for type C infectious diseases (*P* < 0.05). Futhermore, the difference between reported and predicted values was more negative than − 50% for type C infectious diseases in February- August, and − 45.82% and − 27.99% differences for types A and B infectious diseases in February-March. In 2021, the difference was on average − 13.60% for types A and B infectious diseases (*P* < 0.05), and − 19.40% for type C infectious diseases (*P* > 0.05).The reported values for type C infectious diseases were higher than the model predictions from January to March, but lower than the model predictions in the following months. Additionally, there has been a reduction to varying degrees in the difference between reported and predicted incidence for all types of infectious diseases in 2021 compared to 2020.

#### Changes in Infectious Diseases with Different Transmission Routes Compared with Predictions

**Fig. 4:**
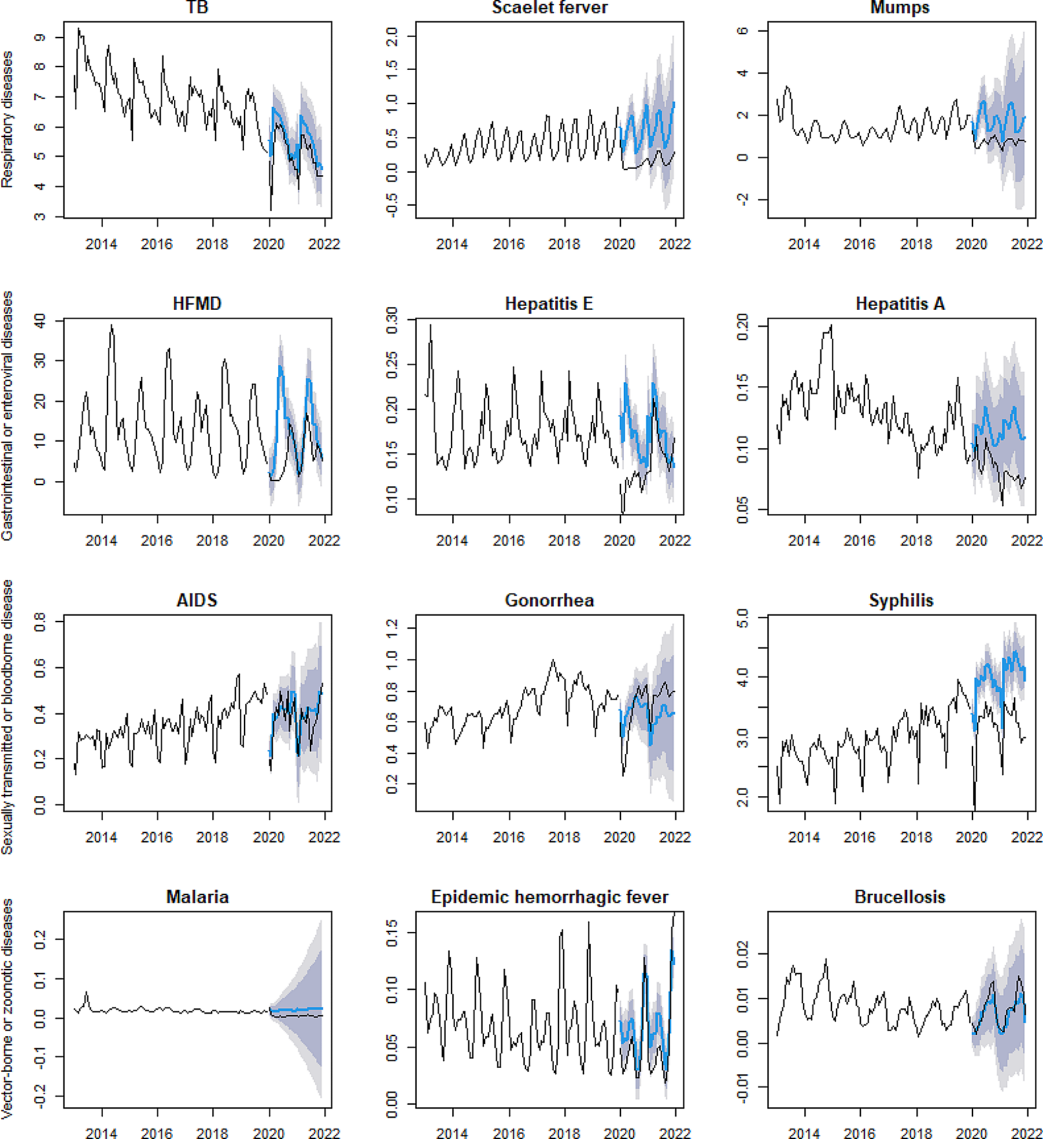
Difference between reported and predicted incidences for infectious diseases with different transmission routes. The black lines represent reported values. The blue line represents predicted values

The results of the subgroup analysis are presented in Fig. 4. For respiratory diseases, TB, scarlet fever, and mumps were included in the analysis. The reported and predicted values for scarlet fever and mumps showed significant differences, with scarlet fever showing the largest difference of -82.13% in 2020 and − 72.85% in 2021. Among the gastrointestinal or enteroviral diseases, HFMD, hepatitis E and hepatitis A were included in the analysis. The difference between the reported and predicted incidence of HFMD in 2020 was − 66.02%, the difference between the reported and predicted incidence of hepatitis E in 2020 was − 32.56%, and the difference between the reported and predicted incidence of hepatitis A in 2020 and 2021 was − 21.32% and − 35.08% respectively. In 2021, the incidence rate of gonorrhea was 22.12% higher than predicted. The reported incidence rates of syphilis in 2020 and 2021 were lower than the predicted incidence rates, with differences of −19.47% and − 22.58%, respectively. The difference between the reported and predicted incidence of malaria in 2020 and 2021 was − 62.29% and − 76.27%, respectively. Details of the model fit and the reported and predicted values of the incidence rates are shown in Appendix 6.

## Discussion

We found that before the COVID-19 pandemic, the incidence of three types of notifiable infectious diseases exhibited limited variation. Specifically, the incidence of type C infectious diseases showed a significant upward trend, while the incidence of types A and B infectious diseases remained relatively stable. However, the annual incidence of three types of infectious diseases decreased significantly after the outbreak of the COVID-19 pandemic. In 2020, the incidence rate of three types of infectious diseases began to decline, with a 15.12% reduction for types A and B infectious disease and a 45.59% reduction for type C infectious diseases. Although there was a slight increase in 2021, it was still lower than the incidence between 2013 and 2019. In addition, there are significant differences in reported and predicted incidence rates of the types of infectious diseases in 2020, and the differences converged in 2021. There is also a significant decrease in respiratory diseases, gastrointestinal or enteroviral diseases, sexually transmitted or bloodborne diseases, and vector-borne or zoonotic diseases during the pandemic.

The reasons for the reduction in the dynamics of notifiable diseases are multifactorial [24]. On the one hand, there may be an issue of underreporting of infectious diseases at the beginning of the pandemic. During the COVID-19 pandemic, the availability and accessibility of medical diagnostic tests for patients were reduced due to the stringent interventions taken by the government. Coupled with public fear [25], this would result in some cases of infectious diseases not being effectively reported. Moreover, compared to some type B infectious diseases, type C infectious diseases are self-limiting and can be cured gradually through patients’ own immunity. The willingness of patients to seek medical treatment decreased during the COVID-19 pandemic and thus the cases were not reported [26], resulting in a higher reduction in the incidence rate of type C infectious diseases during the COVID-19 period than that of types A and B infectious diseases. The aforementioned factors may be partially accountable for the decrease in the incidence of notifiable infectious disease.

On the other hand, following the outbreak of the COVID-19 pandemic, the Chinese government implemented nationwide public health interventions such as using masks, social distancing, and compulsory quarantine. These measures effectively controlled the spread of the COVID-19 pandemic and prevented the transmission of other infectious diseases, resulting in a reduction in their incidence. As COVID-19 was still prevalent in mainland China in 2021, and nationwide public health interventions were still in place, all healthcare facilities, schools, businesses, and many commercial and recreational activities had returned to normal [4]. Leveraging the China Information System for Disease Control and Prevention (CISDCP), one of the world’s largest online reporting platforms for infectious diseases, the 2021 data utilized in this study helps to mitigate the bias stemming from underreporting in research outcomes [13, 27]. Although the incidence rates of all three types of infectious disease increased to some extent in 2021 compared with 2020, the incidence rates of the different types of infectious disease in 2021 were still significantly lower than pre-pandemic levels. Therefore, our findings show that the public health interventions implemented for COVID-19 had a positive impact on other infectious diseases. Such a finding is consistent with studies conducted in various regions and countries globally [28–30].

The study found that the changes in the difference between reported and predicted values were closely associated with alterations in the intensity of public health interventions with the development of the pandemic.For example, the difference between reported and predicted incidences for three types of notifiable infectious diseases reached a maximum negative value of -53.34% in May 2020, after which it began to contract. For types A and B, the difference was − 45.82% in February 2020. Meanwhile, for type C infectious diseases, the maximum negative value reached − 78.93% in May 2020, after which it also began to contract.This is probably due to the fact that after the Wuhan government declared the city blockade on 23 January 2020, the Chinese government imposed a nationwide highest level of emergency response. As a result, all cities closed their schools, 64.3% of cities banned public gatherings and closed entertainment venues, and 64% of cities banned intercity travel [31]. Subsequently, on 8 April 2020, the Chinese government officially announced the lifting of the blockade. China’s pandemic prevention and control shifted to normalized prevention and control phase, and the intensity of related interventions began to decrease. In addition, the reported incidence rates of type C infectious diseases were higher than the model predictions for three consecutive months from January to March 2021. This increase may be related to the winter holidays and the Spring Festival period in China. During this time, there was a slackening of public health interventions due to the large-scale movement of population and the concentration of people in the country [32].

In subgroup analyses of transmission routes, we found that TB, scarlet fever, and mumps selected for respiratory diseases, all showed a decrease during the COVID-19 period. The difference in the incidence rate of TB was − 8.87% and − 9.04%, respectively, similar to the − 11.6% reduction in TB cases during the COVID-19 period in Germany [24]. Similarly, gastrointestinal or enteroviral diseases had a significant reduction in incidence in 2020 and 2021. The finding is consistent with what was found in Zhou’s study [33]. Public health interventions, such as wearing masks and maintaining social distance, are effective in combating respiratory infections transmitted through contact, droplets, and aerosols [34]. Additionally, during the COVID-19 outbreak, a large number of commercial facilities were closed and opportunities for congregate meals were reduced. Therefore, nationwide public health interventions implemented during the outbreak reduced both respiratory infections and gastrointestinal or enteroviral diseases.

For sexually transmitted or blood-borne diseases, there was no significant decrease in the differences between reported and predicted values for HIV and gonorrhoea. The difference for syphilis in 2020 and 2021 was − 19.47% and − 22.58%, respectively (*P* < 0.05) Similar to the findings of Crane et al. who found the least reduction in sexually transmitted diseases and the most reduction in respiratory infections during COVID-19 pandemic [29]. The plausible decrease of sexually relations during the COVID-19 pandemic may partly explain the decreased in the incidence of sexually transmitted or blood-borne diseases [35, 36]. Among the vector-borne or zoonotic diseases, the difference between the reported and predicted incidence rates of malaria, epidemic haemorrhagic fever and brucellosis was also statistically significant. The prevention and control measures taken to restrict the movement of people during the COVID-19 pandemic reduced their exposure to vectors and animal hosts of vector-borne or zoonotic diseases and effectively reduced the incidence of such infectious diseases [4].

## Limitations

Several limitations should be acknowledged in this study. Firstly, there is potential for underreporting of infectious diseases during the COVID-19 period, particularly at the beginning of the pandemic. Due to reduced availability and accessibility of medical treatment, coupled with public fear, some cases of infectious diseases may not be reported to the national system. Despite the potential underreporting in 2020, our data in 2021, which is less affected by the public fears of infectious diseases and thus less subjective to the underreporting issue, also shows a reduction in the incidence of infectious diseases, providing more valid findings. Secondly, various factors affect the occurrence of infectious diseases (e.g. policies & regulations, migration, and vaccination), and this study, which did not use transmission dynamic models for the prediction, failed to take into account the impact of such factors on the incidence of infectious diseases. However, due to the pandemic of COVID-19, it is unlikely that the reduction of the incidence is due to the change of such factors (e.g. improved coverage of vaccinations). Instead, the reduction in incidence is largely attributable to the control measures during the COVID-19 pandemic. Thirdly, this study, with data until December 2021, could not assess the impact of COVID-19 measures in the post-COVID-19 period. Using transmission dynamic models and with the data available for the post-COVID-19 period in the future would address this concern.

## Conclusion

Our findings suggested that the incidence of all types of infectious diseases in China declined significantly during the COVID-19 pandemic, not solely because of the decline in availability and accessibility of medical care and underreporting of infectious diseases, but likely owing to public health interventions that effectively interrupted the transmission of all types of infectious diseases and influenced the epidemic patterns of other infectious diseases. The COVID-19 pandemic posed a significant burden on the national public health system and society, but it served as a wake-up call for increasing public health awareness and investing in public health system. Although national public health measures have been suspended, it is important for the state to strengthen its surveillance of all kinds of infectious diseases and for the local government to increase publicity related to the prevention and control of infectious diseases according to their geographical and seasonal characteristics, and it is recommended that the public maintain good health habits to keep away from infectious diseases.

## Electronic Supplementary Material

Below is the link to the electronic supplementary material.

Supplementary Material 1

## Data Availability

All of data were open accessed from the official website of the Health Commission of the PRC.

## Abbreviations

SARS: Severe acute respiratory syndrome
COVID-19: Corona virus disease 2019
CISDCP: China Information System for Disease Control and Prevention
HFMD: Hand food and mouth disease
AIDS: Acquired immunodeficiency syndrome
ARIMA: Autoregressive integrated moving average
SARIMA: Seasonal autoregressive integrated moving average
ADF: Augmented Dickey-Fuller
AIC: Akaike information criterion
BIC: Bayesian information criterion
RMSE: Root mean square error
MAE: Mean absolute error
MAPE: Mean absolute percentage error
ACF: Autocorrelation function
PACF: Partial autocorrelation function
Q-Q plot: Quantile Quantile plot
TB: Tuberculosis
HIV: Human immunodeficiency virus

## References

[1] Standing up to infectious disease [J]. Nat Microbiol. 2019;4(1):1. 30546101 10.1038/s41564-018-0331-3 PMC7097104

[2] Mahase E. Covid-19: WHO declares pandemic because of alarming levels of spread, severity, and inaction [J]. BMJ. 2020;368:m1036. 32165426 10.1136/bmj.m1036

[3] Li K, Rui J, Song W, et al. Temporal shifts in 24 notifiable infectious diseases in China before and during the COVID-19 pandemic [J]. Nat Commun. 2024;15(1):3891. 38719858 10.1038/s41467-024-48201-8 PMC11079007

[4] Geng MJ, Zhang HY, Yu LJ, et al. Changes in notifiable infectious disease incidence in China during the COVID-19 pandemic [J]. Nat Commun. 2021;12(1):6923. 34836947 10.1038/s41467-021-27292-7 PMC8626444

[5] Barnett-Howell Z, Watson OJ, Mobarak AM. The benefits and costs of social distancing in high- and low-income countries [J]. Trans R Soc Trop Med Hyg. 2021;115(7):807–19. 33440007 10.1093/trstmh/traa140 PMC7928561

[6] Maringe C, Spicer J, Morris M, et al. The impact of the COVID-19 pandemic on cancer deaths due to delays in diagnosis in England, UK: a national, population-based, modelling study [J]. Lancet Oncol. 2020;21(8):1023–34. 32702310 10.1016/S1470-2045(20)30388-0 PMC7417808

[7] Patiño-Lugo D F, Vélez M, Velásquez Salazarp, et al. Non-pharmaceutical interventions for containment, mitigation and suppression of COVID-19 infection [J]. Colomb Med (Cali). 2020;51(2):e4266. 10.25100/cm.v51i2.4266 PMC7518730 33012884

[8] Lai S, Ruktanonchai NW, Zhou L, et al. Effect of non-pharmaceutical interventions to contain COVID-19 in China [J]. Nature. 2020;585(7825):410–3. 32365354 10.1038/s41586-020-2293-x PMC7116778

[9] Feng L, Zhang T, Wang Q, et al. Impact of COVID-19 outbreaks and interventions on influenza in China and the United States [J]. Nat Commun. 2021;12(1):3249. 34059675 10.1038/s41467-021-23440-1 PMC8167168

[10] Olsen SJ, Azziz-Baumgartner E, Budd AP, et al. Decreased influenza activity during the COVID-19 pandemic-United States, Australia, Chile, and South Africa, 2020 [J]. Am J Transplant. 2020;20(12):3681–5. 10.1111/ajt.16381 PMC7753605 33264506

[11] Zhao Z, Zheng C, Qi H, et al. Impact of the coronavirus disease 2019 interventions on the incidence of hand, foot, and mouth disease in mainland China [J]. Lancet Reg Health West Pac. 2022;20:100362. 35005671 10.1016/j.lanwpc.2021.100362 PMC8720138

[12] Lim JT, Dickens BSL, Chew LZX, et al. Impact of sars-cov-2 interventions on dengue transmission [J]. PLoS Negl Trop Dis. 2020;14(10):e0008719. 33119609 10.1371/journal.pntd.0008719 PMC7595279

[13] Zheng J, Zhang N, Shen G, et al. Spatiotemporal and seasonal trends of Class A and B Notifiable Infectious diseases in China: retrospective analysis [J]. JMIR Public Health Surveill. 2023;9:e42820. 37103994 10.2196/42820 PMC10176137

[14] Flaxman S, Mishra S, Gandy A, et al. Estimating the effects of non-pharmaceutical interventions on COVID-19 in Europe [J]. Nature. 2020;584(7820):257–61. 32512579 10.1038/s41586-020-2405-7

[15] Bai BK, Jiang QY, Hou J. The COVID-19 epidemic and other notifiable infectious diseases in China [J]. Microbes Infect. 2022;24(1):104881. 34419605 10.1016/j.micinf.2021.104881 PMC8375246

[16] Chen B, Wang M, Huang X, et al. Changes in incidence of notifiable infectious diseases in China under the Prevention and Control measures of COVID-19 [J]. Front Public Health. 2021;9:728768. 34722440 10.3389/fpubh.2021.728768 PMC8553983

[17] Prevention CCFDCA. Overview of the national epidemic of notifiable infectious diseases in August 2021 [Z]. 2021.

[18] Yang S, Wu J, Ding C, et al. Epidemiological features of and changes in incidence of infectious diseases in China in the first decade after the SARS outbreak: an observational trend study [J]. Lancet Infect Dis. 2017;17(7):716–25. 28412150 10.1016/S1473-3099(17)30227-X PMC7164789

[19] Luo T, Zhou J, Yang J, et al. Early warning and prediction of Scarlet Fever in China using the Baidu Search Index and Autoregressive Integrated moving average with explanatory variable (ARIMAX) Model: Time Series Analysis [J]. J Med Internet Res. 2023;25:e49400. 37902815 10.2196/49400 PMC10644180

[20] Wang C, Li Y, Feng W et al. Epidemiological Features and Forecast Model Analysis for the morbidity of Influenza in Ningbo, China, 2006–2014 [J]. Int J Environ Res Public Health, 2017, 14(6). 10.3390/ijerph14060559 PMC5486245 28587073

[21] Chen Y, Leng K, Lu Y, et al. Epidemiological features and time-series analysis of influenza incidence in urban and rural areas of Shenyang, China, 2010–2018 [J]. Epidemiol Infect. 2020;148:e29. 32054544 10.1017/S0950268820000151 PMC7026897

[22] Ndeh NT, Tesfaldet YT, Budnard J, et al. The secondary outcome of public health measures amidst the COVID-19 pandemic in the spread of other respiratory infectious diseases in Thailand [J]. Travel Med Infect Dis. 2022;48:102348. 35523394 10.1016/j.tmaid.2022.102348 PMC9065650

[23] Hamed KH. Trend detection in hydrologic data: the Mann–Kendall trend test under the scaling hypothesis [J]. J Hydrol. 2008;349(3):350–63.

[24] Ullrich A, Schranz M, Rexroth U, et al. Impact of the COVID-19 pandemic and associated non-pharmaceutical interventions on other notifiable infectious diseases in Germany: an analysis of national surveillance data during week 1-2016 - week 32-2020 [J]. Lancet Reg Health Eur. 2021;6:100103. 34557831 10.1016/j.lanepe.2021.100103 PMC8454829

[25] Tuczyńska M, Matthews-Kozanecka M, Baum E. Accessibility to Non-COVID Health Services in the World during the COVID-19 pandemic: review [J]. Front Public Health. 2021;9:760795. 34976922 10.3389/fpubh.2021.760795 PMC8716399

[26] Czeisler M, Marynak K, Clarke KEN, et al. Delay or Avoidance of Medical Care because of COVID-19-Related concerns - United States, June 2020 [J]. MMWR Morb Mortal Wkly Rep. 2020;69(36):1250–7. 32915166 10.15585/mmwr.mm6936a4 PMC7499838

[27] Zhang H, Wang L, Lai S, et al. Surveillance and early warning systems of infectious disease in China: from 2012 to 2014 [J]. Int J Health Plann Manage. 2017;32(3):329–38. 28632912 10.1002/hpm.2434

[28] Adegbija O, Walker J, Smoll N et al. Notifiable diseases after implementation of COVID-19 public health prevention measures in Central Queensland, Australia [J]. Commun Dis Intell (2018), 2021, 45. 10.33321/cdi.2021.45.11 33632091

[29] Crane MA, Popovic A, Panaparambil R, et al. Reporting of infectious diseases in the United States during the Coronavirus Disease 2019 (COVID-19) pandemic [J]. Clin Infect Dis. 2022;74(5):901–4. 34097015 10.1093/cid/ciab529 PMC8344600

[30] Zhang W, Wu Y, Wen B, et al. Non-pharmaceutical interventions for COVID-19 reduced the incidence of infectious diseases: a controlled interrupted time-series study [J]. Infect Dis Poverty. 2023;12(1):15. 36895021 10.1186/s40249-023-01066-3 PMC9996566

[31] Tian H, Liu Y, Li Y, et al. An investigation of transmission control measures during the first 50 days of the COVID-19 epidemic in China [J]. Science. 2020;368(6491):638–42. 32234804 10.1126/science.abb6105 PMC7164389

[32] Chen J, Feng ZH, Ye L, et al. Travel rush during Chinese Spring Festival and the 2019-nCoV [J]. Travel Med Infect Dis. 2020;37:101686. 32334086 10.1016/j.tmaid.2020.101686 PMC7194668

[33] Zhou X, Qian K, Zhu C, et al. Surveillance, epidemiology, and impact of the coronavirus disease 2019 interventions on the incidence of enterovirus infections in Nanchang, China, 2010–2022 [J]. Front Microbiol. 2023;14:1251683. 37920267 10.3389/fmicb.2023.1251683 PMC10618362

[34] Talic S, Shah S, Wild H, et al. Effectiveness of public health measures in reducing the incidence of covid-19, SARS-CoV-2 transmission, and covid-19 mortality: systematic review and meta-analysis [J]. BMJ. 2021;375:e068302. 34789505 10.1136/bmj-2021-068302 PMC9423125

[35] Sentís A, Prats-Uribe A, López-Corbeto E, et al. The impact of the COVID-19 pandemic on sexually transmitted infections surveillance data: incidence drop or artefact? [J]. BMC Public Health. 2021;21(1):1637. 34493244 10.1186/s12889-021-11630-x PMC8423330

[36] Hammoud MA, Maher L, Holt M, et al. Physical distancing due to COVID-19 disrupts sexual behaviors among Gay and Bisexual men in Australia: implications for trends in HIV and other sexually transmissible infections [J]. J Acquir Immune Defic Syndr. 2020;85(3):309–15. 32740374 10.1097/QAI.0000000000002462

