# Supplementary material for: Impact of the COVID-19 Pandemic on the Incidence of Notifiable Infectious Diseases in China Based on SARIMA Models Between 2013 and 2021
Source: J Epidemiol Glob Health. 2024 Jul 30;14(3):1191–201. doi: 10.1007/s44197-024-00273-x (PMC11442807; doi:10.1007/s44197-024-00273-x)
Supplement: Supplementary file 1 — Supplementary Material 1 [file 44197_2024_273_MOESM1_ESM.docx]

**Appendix 1**

**
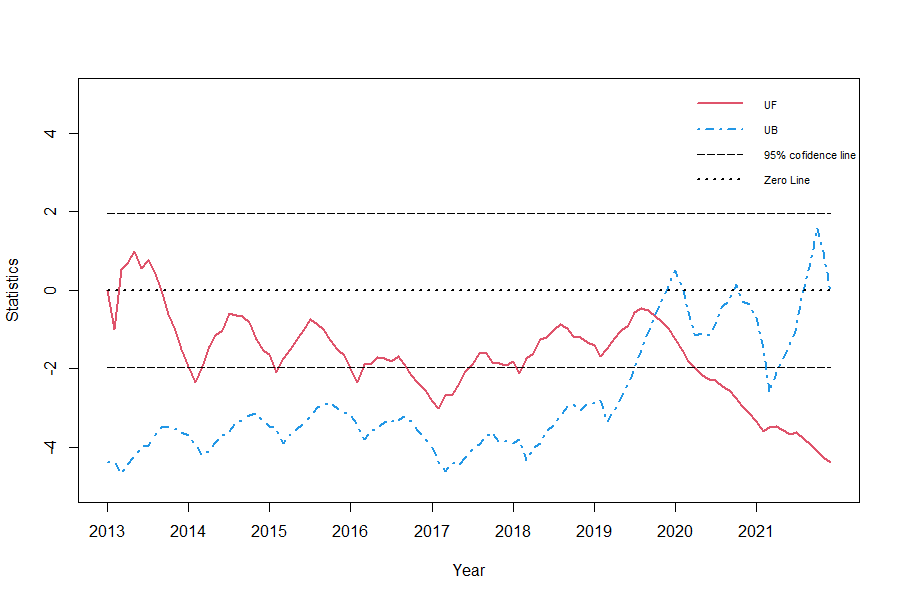
**

**Figure 1. The change point in the incidence of types A and B infectious diseases obtained by the Mann-Kendall mutation test**

**
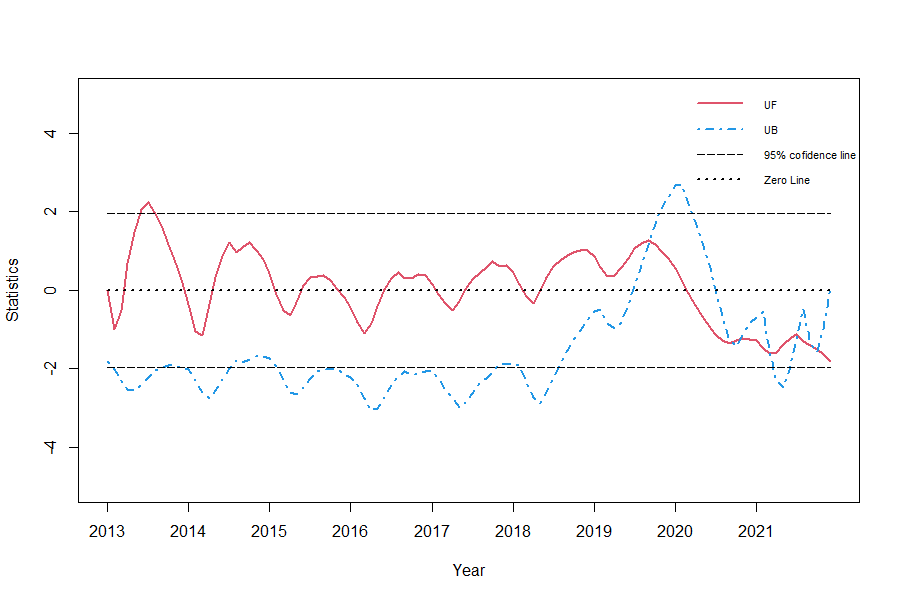
**

**Figure 2. The change point in the incidence of type C infectious diseases obtained by the Mann-Kendall mutation test**

**Appendix 2**

| **Table 1. Optimal ARIMA models for three types of notifiable infectious diseases（including influenza）, and evaluation of the model fit and diagnosis** | | | | | | | | | | |
| --- | --- | --- | --- | --- | --- | --- | --- | --- | --- | --- |
| **Model ID** | **Model Type** | **ADF**  **P value** | **AIC** | **BIC** | **Ljung-Box test** | | **RMSE** | **MAE** | **MAPE** | **R^2^** |
|  |  |  |  |  | Chi-square | P value |  |  |  |  |
| Types A B and C | ARIMA (0,1,2) (1,0,0) _(12)_ | 0.01 | 630.26 | 639.93 | 0.05 | 0.83 | 9.78 | 5.50 | 10.41 | 0.51 |
| Type C | ARIMA (2,1,2) (2,0,0) _(12)_ | 0.01 | 621.98 | 638.91 | 0.57 | 0.45 | 8.94 | 5.00 | 18.68 | 0.55 |

**Figure 1. ARIMA (0,1,2) (1,0,0) _(12)_ model fit, diagnostic and predictive results for the incidence rates of three types of infectious diseases and type C infectious diseases**

(a) Model prediction results. Black are the true values of the training sets and blue indicates the predicted values

(b) Normal Q-Q plot. The solid black line represents the theoretical quantile line

(c) Residual ACF plot. The blue dashed lines indicate the confidence interval

(d) Residual PACF plot. The blue dashed lines indicate the confidence interval


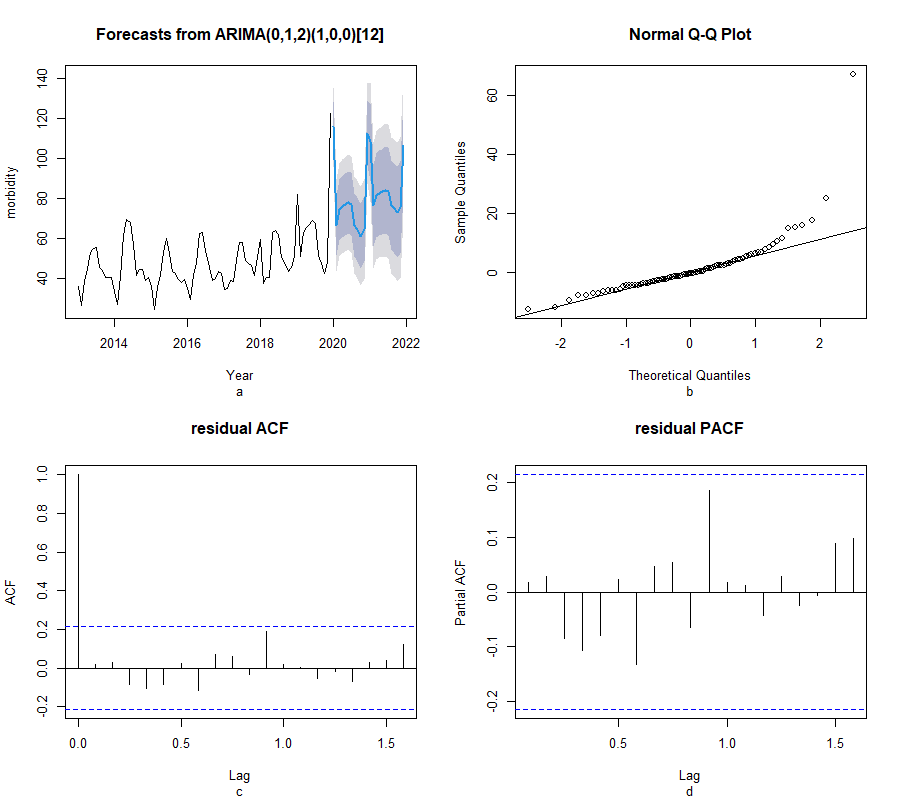


Type A B and C


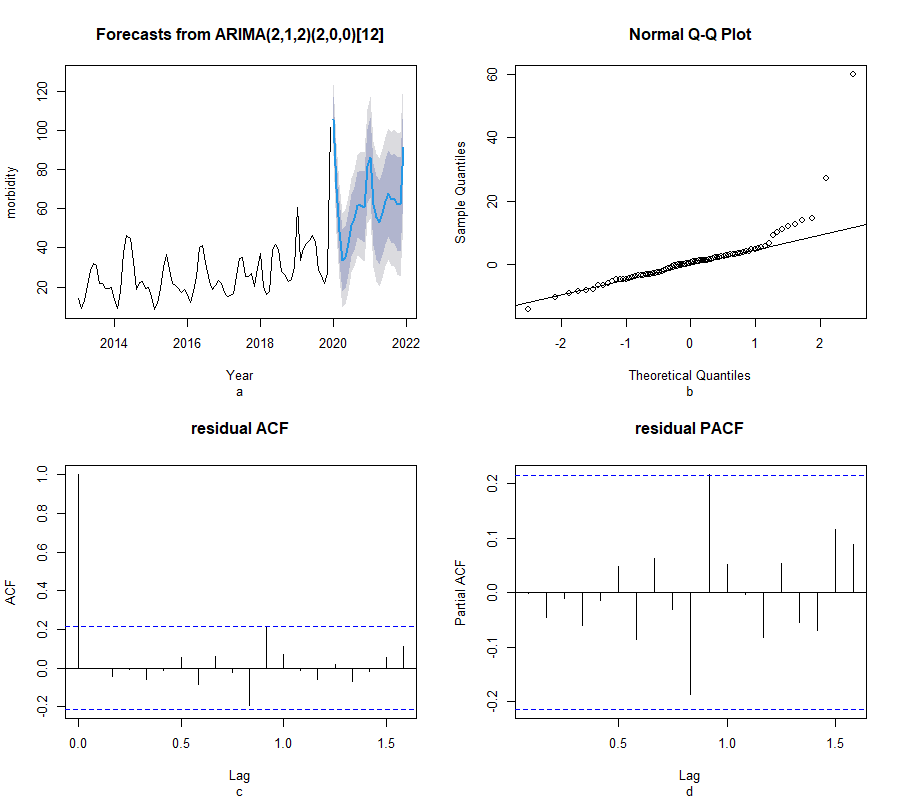


Type C


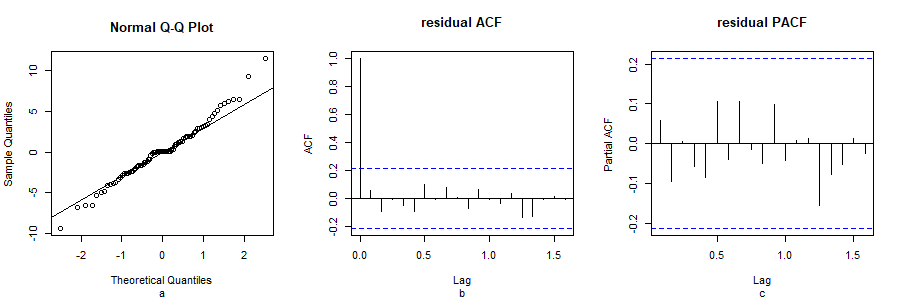
**Appendix 3**

**Figure 1. ARIMA (1,0,0) (1,1,0) _(12)_ model fitting and diagnostic results for the incidence of types A, B and C infectious diseases (Remove Influenza)**

(a) Normal Q-Q plot. The solid black line represents the theoretical quantile line.

(b) Residual ACF plot. The blue dashed lines indicate the confidence interval.


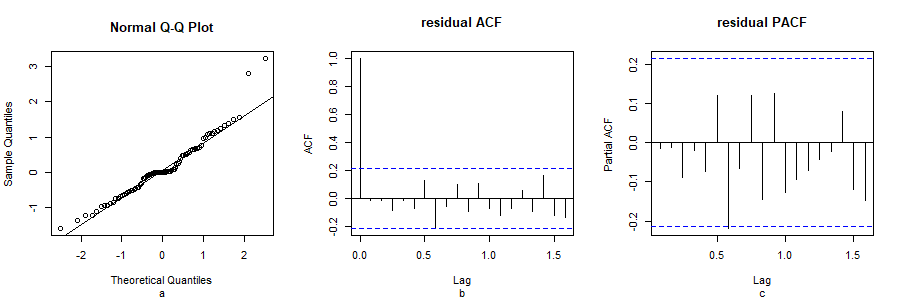
(c) Residual PACF plot. The blue dashed lines indicate the confidence interval.


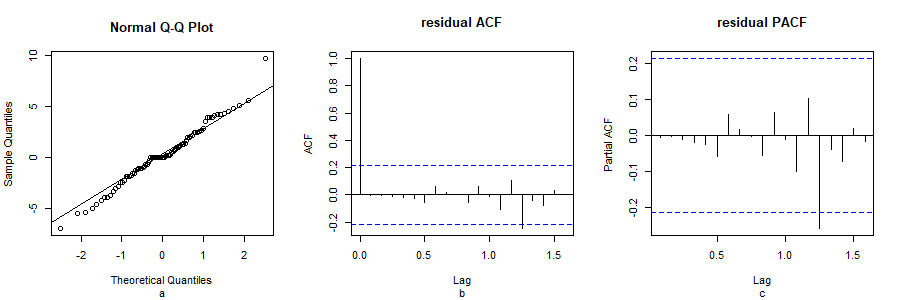
**Figure 2.** **ARIMA (2,1,1) (2,1,0) (12) model fit and diagnostic results for incidence of types A and B infectious disease**

**Figure 3. ARIMA (2,0,0) (1,1,0) (12) model fit and diagnostic results for incidence of type C infectious disease (Remove Influenza)**

**
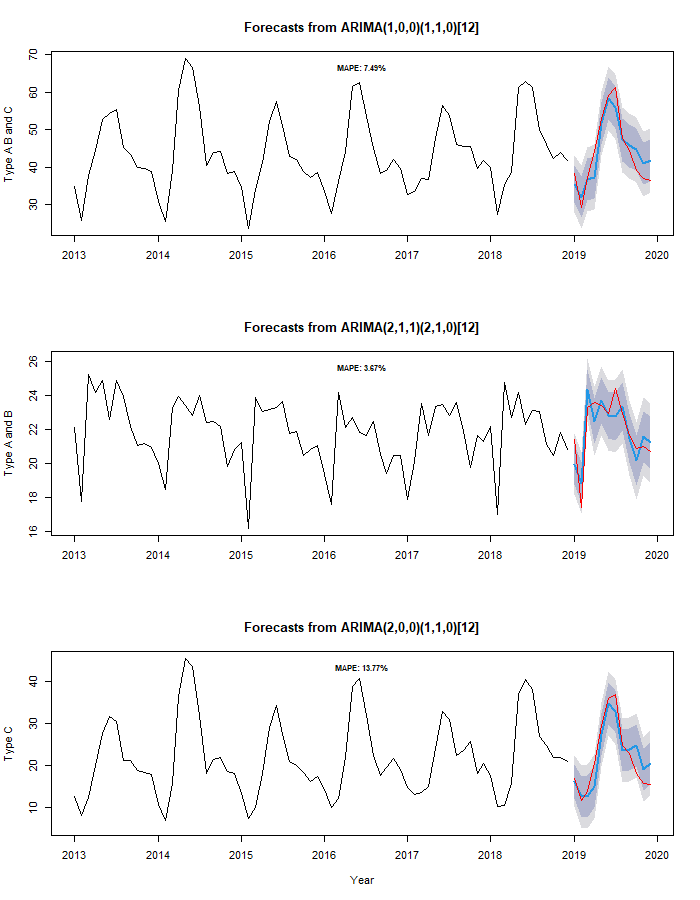
Appendix 4**

**Figure 1 Model validation for model 1, model 2 and model 3. The reported values in 2019 are represented by red, while the predicted values are indicated by blue.**

**Appendix 5**

| **Table 1. Difference between reported and predicted incidence rates of different types of notifiable infectious diseases, 2020-2021** | | | | | | | | | |
| --- | --- | --- | --- | --- | --- | --- | --- | --- | --- |
| **Time** | **Types A B and C** | | **Difference** | **Types A and B** | | **Difference** | **Type C** | | **Difference** |
|  | **Reported** | **Predicted** |  | **Reported** | **Predicted** |  | **Reported** | **Predicted** |  |
| Jan 2020 | 29.73 | 36.68 | -18.93% | 17.54 | 21.01 | -16.52% | 12.19 | 14.67 | -16.87% |
| Feb 2020 | 14.25 | 26.66 | -46.58% | 10.04 | 18.52 | -45.82% | 4.21 | 10.01 | -57.96% |
| Mar 2020 | 21.53 | 35.43 | -39.24% | 17.24 | 23.95 | -27.99% | 4.28 | 11.79 | -63.69% |
| Apr 2020 | 25.10 | 40.07 | -37.35% | 20.06 | 23.04 | -12.92% | 5.04 | 17.48 | -71.17% |
| May 2020 | 27.23 | 58.35 | -53.34% | 19.85 | 23.85 | -16.77% | 7.38 | 35.03 | -78.93% |
| Jun 2020 | 30.25 | 61.52 | -50.83% | 20.56 | 23.25 | -11.57% | 9.69 | 39.32 | -75.36% |
| Jul 2020 | 32.66 | 61.21 | -46.65% | 21.43 | 23.79 | -9.93% | 11.23 | 37.76 | -70.25% |
| Aug 2020 | 32.96 | 49.16 | -32.95% | 20.06 | 23.50 | -14.65% | 12.91 | 26.22 | -50.78% |
| Sep 2020 | 36.87 | 45.52 | -18.99% | 20.54 | 21.90 | -6.20% | 16.33 | 24.30 | -32.78% |
| Oct 2020 | 39.50 | 41.34 | -4.45% | 18.43 | 20.64 | -10.72% | 21.07 | 20.86 | 0.99% |
| Nov 2020 | 40.00 | 41.55 | -3.74% | 19.22 | 21.76 | -11.67% | 20.77 | 20.23 | 2.70% |
| Dec 2020 | 37.01 | 40.05 | -7.60% | 18.83 | 21.25 | -11.42% | 18.18 | 19.47 | -6.65% |
| Jan 2021 | 35.30 | 37.79 | -6.58% | 17.57 | 21.88 | -19.67% | 17.73 | 16.26 | 9.02% |
| Feb 2021 | 26.80 | 28.35 | -5.47% | 14.79 | 17.93 | -17.53% | 12.01 | 11.25 | 6.74% |
| Mar 2021 | 35.51 | 36.71 | -3.26% | 21.99 | 24.40 | -9.88% | 13.52 | 13.37 | 1.14% |
| Apr 2021 | 40.34 | 42.82 | -5.78% | 21.23 | 23.34 | -9.03% | 19.11 | 19.68 | -2.88% |
| May 2021 | 46.57 | 54.80 | -15.01% | 20.50 | 24.16 | -15.16% | 26.07 | 31.21 | -16.47% |
| Jun 2021 | 45.61 | 59.84 | -23.77% | 20.36 | 23.08 | -11.77% | 25.25 | 37.02 | -31.81% |
| Jul 2021 | 42.70 | 61.23 | -30.27% | 21.52 | 23.97 | -10.21% | 21.18 | 37.11 | -42.94% |
| Aug 2021 | 32.50 | 48.16 | -32.52% | 19.34 | 23.48 | -17.62% | 13.16 | 25.18 | -47.73% |
| Sep 2021 | 32.88 | 44.85 | -26.70% | 19.20 | 21.82 | -12.01% | 13.68 | 23.27 | -41.23% |
| Oct 2021 | 33.19 | 39.91 | -16.83% | 17.60 | 20.93 | -15.91% | 15.59 | 19.04 | -18.15% |
| Nov 2021 | 32.11 | 38.44 | -16.45% | 18.49 | 21.89 | -15.54% | 13.62 | 17.17 | -20.66% |
| Dec 2021 | 31.47 | 37.59 | -16.28% | 19.36 | 21.24 | -8.83% | 12.11 | 16.79 | -27.88% |

**Appendix 6**

| **Table 1. Optimal ARIMA models for different transmission route, and evaluation of the model fit and diagnosis** | | | | | | | | | | |
| --- | --- | --- | --- | --- | --- | --- | --- | --- | --- | --- |
| **Model ID** | **Model Type** | **ADF**  **P value** | **AIC** | **BIC** | **Ljung-Box test** | | **RMSE** | **MAE** | **MAPE** | **R^2^** |
|  |  |  |  |  | Chi-square | P value |  |  |  |  |
| *Respiratory diseases* | | | | | | | | | | |
| TB | ARIMA (0,1,1) (2,1,0) _(12)_ | 0.01 | 80.05 | 89.1 | 13.26 | ＜0.001 | 0.35 | 0.26 | 3.92 | 0.84 |
| Scarlet fever | ARIMA (1,1,1) (0,1,2) _(12)_ | 0.01 | -189.43 | -178.1 | 10.56 | 0.001 | 0.05 | 0.04 | 11.66 | 0.94 |
| Mumps | ARIMA (0,1,1) (0,1,0) _[12]_ | 0.01 | -29.69 | -25.34 | 22.06 | ＜0.001 | 0.17 | 0.11 | 9.50 | 0.92 |
| *Gastrointestinal or enteroviral diseases* | | | | | | | | | | |
| HFMD | ARIMA (2,0,0) (1,1,0) _(12)_ | 0.01 | 379.82 | 388.93 | 46.06 | ＜0.001 | 2.76 | 1.92 | 18.55 | 0.90 |
| Hepatitis E | ARIMA (0,0,0) (0,1,0) _(12)_ | 0.01 | -388.96 | -386.68 | 28.16 | ＜0.001 | 0.01 | 0.01 | 5.97 | 0.82 |
| Hepatitis A | ARIMA (1,1,1) (2,0,0) _(12)_ | 0.01 | -457.57 | -445.48 | 4.33 | 0.034 | 0.01 | 0.01 | 8.45 | 0.71 |
| *Sexually transmitted or bloodborne diseases* | | | | | | | | | | |
| AIDS | ARIMA (1,0,0) (0,1,1) _(12)_ | 0.01 | -269.56 | -262.78 | 3.68 | 0.055 | 0.03 | 0.02 | 7.35 | 0.89 |
| Gonorrhea | ARIMA (2,2,1) (0,1,1) _(12)_ | 0.01 | -223.16 | -211.91 | 9.80 | 0.002 | 0.04 | 0.03 | 3.52 | 0.92 |
| Syphilis | ARIMA (3,1,1) (2,1,0) _(12)_ | 0.01 | -59.87 | -44.03 | 19.61 | ＜0.001 | 0.12 | 0.09 | 3.00 | 0.92 |
| *Vector-borne or zoonotic diseases* | | | | | | | | | | |
| Malaria | ARIMA (5,2,0) (2,0,0) _(12)_ | 0.01 | -584.53 | -565.28 | 13.975 | ＜0.001 | 0.01 | 0.004 | 17.46 | 0.54 |
| Epidemic hemorrhagic fever | ARIMA (1,0,1) (2,1,1) _(12)_ | 0.02 | -439.82 | -426.16 | 22.85 | ＜0.001 | 0.01 | 0.01 | 9.80 | 0.92 |
| Brucellosis | ARIMA (0,1,0) (0,1,1) _(12)_ | 0.01 | -282.73 | -278.20 | 15.86 | ＜0.001 | 0.03 | 0.02 | 7.91 | 0.95 |

| **Table 2.** **Comparison of reported and predicted incidence rates of infectious diseases with different transmission routes in 2020 and 2021** | | | | | | | |
| --- | --- | --- | --- | --- | --- | --- | --- |
| **Transmission route** | **2020** | | | **2021** | | | |
|  | **Fit** | **Difference** | **P value** | **Fit** | **Difference** | **P value** |  |
| *Respiratory diseases* | | | | | | |  |
| TB | 68.70 | -8.87% | 0.114 | 64.48 | -9.04% | 0.078 |  |
| Scarlet fever | 6.88 | -82.13% | < 0.001 | 7.70 | -72.85% | < 0.001 |  |
| Mumps | 20.98 | -55.43% | < 0.001 | 20.24 | -57.72% | < 0.001 |  |
| *Gastrointestinal or enteroviral diseases* | | | | | | |  |
| HFMD | 161.73 | -66.02% | 0.008 | 145.23 | -33.27% | 0.266 |  |
| Hepatitis E | 2.09 | -32.56% | < 0.001 | 2.09 | -9.05% | 0.160 |  |
| Hepatitis A | 1.41 | -21.32% | < 0.001 | 1.35 | -35.08% | < 0.001 |  |
| *Sexually transmitted or bloodborne diseases* | | | | | | |  |
| AIDS | 4.70 | -4.00% | 0.799 | 4.65 | -6.98% | 0.410 |  |
| Gonorrhea | 8.20 | -7.13% | 0.977 | 7.54 | 22.12% | < 0.001 |  |
| Syphilis | 46.38 | -19.47% | < 0.001 | 49.20 | -22.58% | < 0.001 |  |
| *Vector-borne or zoonotic diseases* | | | | | | |  |
| Malaria | 0.22 | -62.29% | < 0.001 | 0.25 | -76.27% | < 0.001 |  |
| Epidemic hemorrhagic fever | 0.78 | -22.22% | 0.068 | 0.85 | -20.69% | 0.039 |  |
| Brucellosis | 3.61 | -0.95% | 0.799 | 3.70 | 40.95% | 0.052 |  |

| **Table 3. Difference between reported and predicted incidence rates of respiratory diseases, 2020-2021** | | | | | | | | | |
| --- | --- | --- | --- | --- | --- | --- | --- | --- | --- |
| **Time** | **TB** | | **Difference** | **Scarlet fever** | | **Difference** | **Mumps** | | **Difference** |
|  | **Reported** | **Predicted** |  | **Reported** | **Predicted** |  | **Reported** | **Predicted** |  |
| Jan 2020 | 4.83 | 5.56 | -13.03% | 0.45 | 0.65 | -30.21% | 1.23 | 1.66 | -26.32% |
| Feb 2020 | 3.21 | 5.02 | -36.06% | 0.04 | 0.29 | -85.47% | 0.57 | 0.81 | -29.31% |
| Mar 2020 | 5.24 | 6.64 | -20.97% | 0.03 | 0.47 | -93.19% | 0.43 | 1.29 | -66.55% |
| Apr 2020 | 6.12 | 6.45 | -5.15% | 0.03 | 0.59 | -94.64% | 0.44 | 2.00 | -77.94% |
| May 2020 | 5.96 | 6.39 | -6.73% | 0.04 | 0.76 | -94.69% | 0.66 | 2.57 | -74.19% |
| Jun 2020 | 6.07 | 6.33 | -4.19% | 0.05 | 0.82 | -94.10% | 0.86 | 2.66 | -67.73% |
| Jul 2020 | 5.94 | 6.09 | -2.46% | 0.06 | 0.51 | -88.87% | 0.76 | 2.05 | -63.07% |
| Aug 2020 | 5.46 | 5.82 | -6.20% | 0.05 | 0.26 | -79.36% | 0.64 | 1.28 | -49.64% |
| Sep 2020 | 5.39 | 5.45 | -1.20% | 0.06 | 0.34 | -81.61% | 0.94 | 1.33 | -28.87% |
| Oct 2020 | 4.85 | 4.94 | -1.89% | 0.08 | 0.45 | -82.61% | 0.92 | 1.51 | -39.38% |
| Nov 2020 | 4.97 | 5.09 | -2.26% | 0.14 | 0.77 | -82.19% | 1.09 | 1.92 | -43.52% |
| Dec 2020 | 4.58 | 4.93 | -7.17% | 0.19 | 0.97 | -80.27% | 0.82 | 1.91 | -57.33% |
| Jan 2021 | 4.59 | 5.40 | -14.92% | 0.18 | 0.71 | -74.99% | 0.50 | 1.60 | -68.69% |
| Feb 2021 | 3.93 | 4.50 | -12.82% | 0.07 | 0.36 | -80.35% | 0.31 | 0.75 | -58.41% |
| Mar 2021 | 5.72 | 6.39 | -10.36% | 0.13 | 0.52 | -74.99% | 0.68 | 1.23 | -44.49% |
| Apr 2021 | 5.71 | 6.10 | -6.40% | 0.20 | 0.66 | -69.83% | 0.81 | 1.94 | -57.91% |
| May 2021 | 5.33 | 6.05 | -11.89% | 0.29 | 0.85 | -65.76% | 0.85 | 2.50 | -66.14% |
| Jun 2021 | 5.23 | 5.82 | -10.10% | 0.30 | 0.90 | -67.12% | 0.85 | 2.59 | -67.18% |
| Jul 2021 | 5.43 | 5.71 | -4.85% | 0.17 | 0.57 | -69.91% | 0.66 | 1.99 | -66.58% |
| Aug 2021 | 4.81 | 5.44 | -11.48% | 0.08 | 0.34 | -76.16% | 0.59 | 1.22 | -51.33% |
| Sep 2021 | 4.80 | 5.07 | -5.32% | 0.09 | 0.41 | -79.07% | 0.89 | 1.26 | -29.40% |
| Oct 2021 | 4.35 | 4.66 | -6.60% | 0.12 | 0.53 | -78.14% | 0.79 | 1.45 | -45.64% |
| Nov 2021 | 4.37 | 4.78 | -8.48% | 0.19 | 0.84 | -76.98% | 0.84 | 1.86 | -54.79% |
| Dec 2021 | 4.38 | 4.58 | -4.40% | 0.28 | 1.03 | -72.53% | 0.77 | 1.85 | -58.36% |

| **Table 4. Difference between reported and predicted incidence rates of Gastrointestinal or enteroviral diseases, 2020-2021** | | | | | | | | | |
| --- | --- | --- | --- | --- | --- | --- | --- | --- | --- |
| **Time** | **HFMD** | | **Difference** | **Hepatitis E** | | **Difference** | **Hepatitis A** | | **Difference** |
|  | **Reported** | **Predicted** |  | **Reported** | **Predicted** |  | **Reported** | **Predicted** |  |
| Jan 2020 | 2.03 | 2.32 | -12.56% | 0.12 | 0.19 | -39.87% | 0.09 | 0.11 | -20.27% |
| Feb 2020 | 0.23 | 1.05 | -77.85% | 0.07 | 0.16 | -54.32% | 0.09 | 0.11 | -13.73% |
| Mar 2020 | 0.20 | 2.71 | -92.43% | 0.12 | 0.23 | -48.95% | 0.11 | 0.12 | -11.49% |
| Apr 2020 | 0.21 | 9.32 | -97.70% | 0.12 | 0.22 | -42.65% | 0.08 | 0.12 | -30.35% |
| May 2020 | 0.28 | 25.68 | -98.91% | 0.11 | 0.19 | -40.99% | 0.08 | 0.12 | -32.17% |
| Jun 2020 | 0.45 | 28.81 | -98.45% | 0.12 | 0.17 | -27.44% | 0.09 | 0.13 | -30.07% |
| Jul 2020 | 1.91 | 26.41 | -92.76% | 0.13 | 0.18 | -25.55% | 0.11 | 0.13 | -19.16% |
| Aug 2020 | 4.22 | 15.56 | -72.85% | 0.13 | 0.17 | -28.03% | 0.10 | 0.12 | -18.97% |
| Sep 2020 | 8.23 | 15.83 | -48.03% | 0.13 | 0.15 | -14.98% | 0.10 | 0.12 | -18.12% |
| Oct 2020 | 14.28 | 13.07 | 9.26% | 0.11 | 0.14 | -22.75% | 0.08 | 0.11 | -22.08% |
| Nov 2020 | 13.50 | 11.66 | 15.80% | 0.12 | 0.15 | -20.78% | 0.09 | 0.11 | -16.82% |
| Dec 2020 | 9.41 | 9.32 | 1.06% | 0.13 | 0.14 | -4.03% | 0.09 | 0.11 | -21.56% |
| Jan 2021 | 4.48 | 5.03 | -10.92% | 0.13 | 0.19 | -32.59% | 0.07 | 0.10 | -34.38% |
| Feb 2021 | 1.31 | 1.77 | -25.66% | 0.13 | 0.16 | -19.85% | 0.05 | 0.10 | -45.68% |
| Mar 2021 | 2.56 | 3.56 | -27.96% | 0.21 | 0.23 | -7.73% | 0.08 | 0.12 | -32.83% |
| Apr 2021 | 10.21 | 10.67 | -4.31% | 0.20 | 0.22 | -8.16% | 0.08 | 0.12 | -31.02% |
| May 2021 | 17.06 | 20.95 | -18.56% | 0.18 | 0.19 | -7.36% | 0.08 | 0.11 | -30.38% |
| Jun 2021 | 15.90 | 25.40 | -37.42% | 0.16 | 0.17 | -4.53% | 0.08 | 0.13 | -38.38% |
| Jul 2021 | 11.54 | 24.88 | -53.60% | 0.15 | 0.18 | -14.09% | 0.07 | 0.13 | -45.02% |
| Aug 2021 | 5.05 | 14.30 | -64.69% | 0.15 | 0.17 | -14.43% | 0.07 | 0.12 | -38.87% |
| Sep 2021 | 6.14 | 14.12 | -56.54% | 0.14 | 0.15 | -5.35% | 0.08 | 0.11 | -30.60% |
| Oct 2021 | 9.35 | 10.37 | -9.81% | 0.13 | 0.14 | -6.10% | 0.07 | 0.10 | -35.41% |
| Nov 2021 | 8.04 | 8.36 | -3.86% | 0.15 | 0.15 | -1.50% | 0.07 | 0.10 | -28.86% |
| Dec 2021 | 5.27 | 5.83 | -9.54% | 0.17 | 0.14 | 24.02% | 0.08 | 0.10 | -27.53% |

| **Table 5. Difference between reported and predicted incidence rates of Sexually transmitted or bloodborne diseases, 2020-2021** | | | | | | | | | | |
| --- | --- | --- | --- | --- | --- | --- | --- | --- | --- | --- |
| **Time** | **AIDS** | | **Difference** | **Gonorrhea** | | **Difference** | **Syphilis** | | **Difference** |  |
|  | **Reported** | **Predicted** |  | **Reported** | **Predicted** |  | **Reported** | **Predicted** |  |  |
| Jan 2020 | 0.20 | 0.24 | -16.80% | 0.59 | 0.68 | -13.06% | 2.83 | 3.40 | -16.57% |  |
| Feb 2020 | 0.15 | 0.22 | -29.36% | 0.25 | 0.50 | -49.46% | 1.53 | 3.11 | -50.80% |  |
| Mar 2020 | 0.34 | 0.40 | -13.39% | 0.33 | 0.63 | -47.44% | 2.94 | 4.00 | -26.45% |  |
| Apr 2020 | 0.43 | 0.38 | 13.27% | 0.45 | 0.64 | -30.07% | 3.34 | 3.86 | -13.48% |  |
| May 2020 | 0.39 | 0.41 | -3.28% | 0.58 | 0.69 | -16.23% | 3.34 | 4.04 | -17.27% |  |
| Jun 2020 | 0.49 | 0.43 | 14.19% | 0.66 | 0.68 | -3.10% | 3.32 | 3.95 | -15.84% |  |
| Jul 2020 | 0.44 | 0.42 | 4.20% | 0.76 | 0.76 | 0.17% | 3.60 | 4.19 | -14.11% |  |
| Aug 2020 | 0.37 | 0.41 | -10.52% | 0.77 | 0.77 | -0.03% | 3.35 | 4.22 | -20.71% |  |
| Sep 2020 | 0.49 | 0.42 | 16.64% | 0.83 | 0.72 | 14.74% | 3.50 | 4.07 | -14.01% |  |
| Oct 2020 | 0.32 | 0.40 | -18.01% | 0.75 | 0.69 | 8.72% | 3.17 | 3.83 | -17.18% |  |
| Nov 2020 | 0.42 | 0.50 | -16.12% | 0.80 | 0.71 | 12.59% | 3.24 | 3.96 | -18.26% |  |
| Dec 2020 | 0.46 | 0.49 | -4.80% | 0.84 | 0.72 | 16.53% | 3.19 | 3.76 | -15.13% |  |
| Jan 2021 | 0.23 | 0.23 | 0.68% | 0.73 | 0.63 | 15.81% | 2.84 | 3.84 | -26.02% |  |
| Feb 2021 | 0.22 | 0.21 | 1.59% | 0.54 | 0.45 | 20.93% | 2.38 | 3.15 | -24.50% |  |
| Mar 2021 | 0.42 | 0.39 | 7.54% | 0.77 | 0.58 | 32.77% | 3.59 | 4.32 | -16.95% |  |
| Apr 2021 | 0.37 | 0.37 | 0.63% | 0.77 | 0.59 | 31.08% | 3.48 | 4.12 | -15.60% |  |
| May 2021 | 0.36 | 0.40 | -10.81% | 0.76 | 0.64 | 19.72% | 3.40 | 4.33 | -21.55% |  |
| Jun 2021 | 0.42 | 0.43 | -1.17% | 0.78 | 0.63 | 23.04% | 3.36 | 4.09 | -17.88% |  |
| Jul 2021 | 0.24 | 0.42 | -42.52% | 0.83 | 0.70 | 18.50% | 3.65 | 4.42 | -17.49% |  |
| Aug 2021 | 0.33 | 0.41 | -18.28% | 0.85 | 0.71 | 19.88% | 3.26 | 4.42 | -26.13% |  |
| Sep 2021 | 0.36 | 0.42 | -15.04% | 0.83 | 0.67 | 24.54% | 3.24 | 4.25 | -23.65% |  |
| Oct 2021 | 0.38 | 0.39 | -3.18% | 0.76 | 0.64 | 19.48% | 2.90 | 4.12 | -29.64% |  |
| Nov 2021 | 0.46 | 0.49 | -6.49% | 0.79 | 0.66 | 20.09% | 2.99 | 4.18 | -28.48% |  |
| Dec 2021 | 0.53 | 0.48 | 9.58% | 0.80 | 0.66 | 21.38% | 3.00 | 3.95 | -24.16% |  |

| **Table 6. Difference between reported and predicted incidence rates of Vector-borne or zoonotic diseases, 2020-2021** | | | | | | | | | |
| --- | --- | --- | --- | --- | --- | --- | --- | --- | --- |
| **Time** | **Malaria** | | **Difference** | **Epidemic hemorrhagic fever** | | **Difference** | **Brucellosis** | | **Difference** |
|  | **Reported** | **Predicted** |  | **Reported** | **Predicted** |  | **Reported** | **Predicted** |  |
| Jan 2020 | 0.0249 | 0.0184 | 35.14% | 0.0489 | 0.0732 | -33.29% | 0.1746 | 0.1890 | -7.60% |
| Feb 2020 | 0.0090 | 0.0182 | -50.63% | 0.0267 | 0.0536 | -50.16% | 0.0666 | 0.1871 | -64.38% |
| Mar 2020 | 0.0066 | 0.0153 | -56.95% | 0.0309 | 0.0581 | -46.78% | 0.2506 | 0.3242 | -22.72% |
| Apr 2020 | 0.0039 | 0.0166 | -76.70% | 0.0386 | 0.0556 | -30.61% | 0.3828 | 0.3605 | 6.21% |
| May 2020 | 0.0029 | 0.0184 | -84.44% | 0.0490 | 0.0713 | -31.32% | 0.3760 | 0.4250 | -11.54% |
| Jun 2020 | 0.0053 | 0.0183 | -71.07% | 0.0590 | 0.0750 | -21.29% | 0.4423 | 0.4384 | 0.90% |
| Jul 2020 | 0.0039 | 0.0194 | -80.08% | 0.0359 | 0.0549 | -34.52% | 0.4598 | 0.4290 | 7.18% |
| Aug 2020 | 0.0041 | 0.0188 | -78.29% | 0.0224 | 0.0316 | -29.17% | 0.3551 | 0.3639 | -2.40% |
| Sep 2020 | 0.0065 | 0.0192 | -66.08% | 0.0229 | 0.0302 | -24.34% | 0.3208 | 0.2575 | 24.59% |
| Oct 2020 | 0.0056 | 0.0181 | -69.29% | 0.0436 | 0.0540 | -19.17% | 0.2290 | 0.1938 | 18.13% |
| Nov 2020 | 0.0045 | 0.0196 | -77.08% | 0.1283 | 0.1139 | 12.64% | 0.2579 | 0.2186 | 18.00% |
| Dec 2020 | 0.0052 | 0.0177 | -70.50% | 0.1043 | 0.1135 | -8.10% | 0.2638 | 0.2269 | 16.28% |
| Jan 2021 | 0.0048 | 0.0213 | -77.37% | 0.0398 | 0.0742 | -46.34% | 0.2249 | 0.1963 | 14.59% |
| Feb 2021 | 0.0041 | 0.0206 | -80.01% | 0.0259 | 0.0498 | -48.05% | 0.2426 | 0.1944 | 24.82% |
| Mar 2021 | 0.0045 | 0.0183 | -75.59% | 0.0300 | 0.0614 | -51.11% | 0.5114 | 0.3315 | 54.28% |
| Apr 2021 | 0.0052 | 0.0190 | -72.46% | 0.0361 | 0.0625 | -42.22% | 0.5559 | 0.3677 | 51.17% |
| May 2021 | 0.0069 | 0.0203 | -66.09% | 0.0485 | 0.0800 | -39.38% | 0.5735 | 0.4323 | 32.66% |
| Jun 2021 | 0.0077 | 0.0213 | -63.79% | 0.0513 | 0.0783 | -34.53% | 0.6850 | 0.4457 | 53.69% |
| Jul 2021 | 0.0065 | 0.0227 | -71.25% | 0.0331 | 0.0578 | -42.81% | 0.6532 | 0.4362 | 49.74% |
| Aug 2021 | 0.0042 | 0.0218 | -80.52% | 0.0181 | 0.0343 | -47.40% | 0.4864 | 0.3711 | 31.06% |
| Sep 2021 | 0.0035 | 0.0220 | -84.21% | 0.0206 | 0.0303 | -32.08% | 0.4202 | 0.2648 | 58.69% |
| Oct 2021 | 0.0033 | 0.0213 | -84.72% | 0.0480 | 0.0608 | -20.96% | 0.2566 | 0.2011 | 27.57% |
| Nov 2021 | 0.0044 | 0.0235 | -81.30% | 0.1502 | 0.1352 | 11.07% | 0.2585 | 0.2258 | 14.45% |
| Dec 2021 | 0.0051 | 0.0217 | -76.45% | 0.1701 | 0.1222 | 39.22% | 0.3484 | 0.2342 | 48.79% |
